# Congenital ptosis with aponeurotic maldevelopment: clinical and surgical perspectives: Aponeurotic maldevelopment in congenital ptosis

**DOI:** 10.1007/s10792-024-03053-5

**Published:** 2024-03-02

**Authors:** Yomna A Alahmadawy, Rania A Ahmed

**Affiliations:** Ophthalmology Department, Faculty of Medicine, Cairo University, Giza, Egypt

**Keywords:** Congenital ptosis, Aponeurotic ptosis, Levator muscle surgery

## Abstract

**Purpose:**

Levator muscle dystrophy has been commonly accused being the main pathology of congenital ptosis, nevertheless, few reports drew attention to the existence of congenital aponeurotic defects. This study aims at highlighting the detailed clinical and surgical features of aponeurotic maldevelopment together with the efficacy of simple aponeurosis repair.

**Methods:**

This is a retrospective nonrandomised study including patients with congenital ptosis who underwent levator muscle surgery throughout 4 years. Patients’ records were reviewed for the preoperative clinical assessment and photographs, intraoperative recorded data, and videos as well as postoperative data and photographs.

**Results:**

Twenty-seven eyes of 22 patients (9.4%) out of 287 eyes were recorded to have absent levator muscle at its typical anatomical insertion site intraoperatively. The mean preoperative MRD1 was (0.44 ± 1.17 mm). The mean levator function was 8.56 ± 3.89 mm, with higher-than-normal crease position (mean value 10.07 ± 1.62 mm). 25 eyes of included cases (92.6%) showed total absence of the levator aponeurosis edge which only was revealed after cutting through the orbital septal covering of the preaponeurotic fat.

**Conclusion:**

Congenital aponeurotic defect is an established yet under reported entity of congenital ptosis with reproducible characteristic intraoperative findings. Simple aponeurosis repair could achieve ptosis correction in such cases.

**Supplementary Information:**

The online version contains supplementary material available at 10.1007/s10792-024-03053-5.

## Introduction

Childhood ptosis is commonly congenital due to levator muscle dystrophy [1]. However, in this age group, it could also be neurogenic, mechanical, or aponeurotic. Aponeurotic disinsertion or dehiscence is a well-recognised cause of involutional or traumatic ptosis [2], which is classically corrected by aponeurotic repair, a technique that was first described by Quickert in 1970 [3]. Paediatric aponeurotic ptosis is commonly acquired; nevertheless, congenital aponeurotic defects do exist but less recognized and usually an overlooked cause of congenital ptosis. Those defects were first described by Anderson and Gordy in 1979 and were attributed to failure of the levator aponeurosis to correctly insert to the anterior surface of the tarsus [4].

The aim of this study is to highlight the incidence of aponeurotic maldevelopment in congenital ptosis, the pre and intraoperative signs. It also focuses on the outcome of aponeurosis surgical repair.

## Methods

This is a retrospective nonrandomised study in which data of patients with congenital ptosis with fair to good levator muscle function who underwent levator muscle surgery by the authors between 2019 and 2022 were retrieved from the hospital records at ophthalmology department at Cairo university hospital.

Data of the preoperative assessment, intraoperative and postoperative data together with recorded photographs and videos were reviewed. Patients with any medical illness, ocular inflammation, trauma, contact lens wearing were excluded. All photos of the included patients were routinely taken by the authors in primary gaze position using a digital camera with flashlight to show the corneal light reflex, in downgaze and during eye closure.

Data included:

1. General ophthalmological assessment (best corrected visual acuity when feasible, pupil, extraocular motility, anterior and posterior segment examination).
2. Ptosis assessment (marginal reflex distance 1 (MRD1), crease height, levator muscle function, Bell’s phenomena, lid position in downward gaze to detect lid lag).

All patients with congenital ophthalmological defects were examined by a geneticist to exclude other congenital anomalies.

A crease incision was done through the skin and the orbicularis muscle. The orbital septum was opened layer by layer slightly higher than the superior tarsal border to avoid accidental injury to the levator aponeurosis till bulging of the pre-aponeurotic fat that was retracted to identify the aponeurosis. Once the muscle was identified at its insertion, levator resection was accomplished according to the Beard’s tables as well as the intraoperative assessment based on Berke’s formula and the surgeon’s experience to reach the desired intraoperative palpebral fissure height.

Cases where the aponeurosis was missing at its anatomical insertion were marked as congenital ptosis with aponeurotic maldevelopment and documented with Intraoperative photographs. Further upward dissection was done till the aponeurosis edge was detected and the distance between it and the tarsus was measured using a calliper. The identified aponeurosis was advanced and reattached to the anterior surface of the upper third of the tarsus using double armed prolene 5/0 suture. Lid height and contour were checked. Skin incision was closed using polyglycolic 6/0 suture with incorporating bites of the aponeurosis. All patients received combined topical antibiotic steroid ointment and intensive lubricants.

The data of the first postoperative day and week included the degree of lid oedema, lagophthalmos and corneal condition. The final MRD1, lid contour and crease height were detected at one month.

**Ethics approval**: This study adhered to international ethical standards and tenets of the Declaration of Helsinki.

This study was approved by the Ethics Review Committee of Cairo University, Faculty of Medicine with ID approval N-98–2023.

**Consent to participate:** Patient’s privacy was respected throughout the study. Participants’ parents were informed about the study, and a written informed consent about using their photos was obtained.

## Statistical analysis

Data analyses using SPSS software (Statistical Package for the Social Sciences, version 24, SSPS Inc, Chicago, IL, USA) was done. Frequency tables with percentages were used for categorical variables while descriptive statistics as (mean and standard deviation) were used for numerical variables. Paired Student-t was used to compare quantitative variables, while McNemar test was used to analyse categorical variables. A p-value < 0.05 is considered statistically significant. Correlation was studied by calculating the Pearson correlation index.

## Results

Files of 287 eyes with history of levator muscle surgery for congenital ptosis were retrieved and reviewed. Twenty-seven eyes of 22 patients (9.4%) were recorded to have absent levator muscle at its anatomical insertion site intraoperatively thus meeting the criteria of congenital aponeurotic ptosis (Table 1). The mean age was 9.48 y ± 6.35 and 40.7% were boys. The condition was bilateral in five patients. None of the included patients had any associated congenital anomalies or family history of congenital ptosis or anomalies.

## Preoperative clinical data

**Fig. 1:**
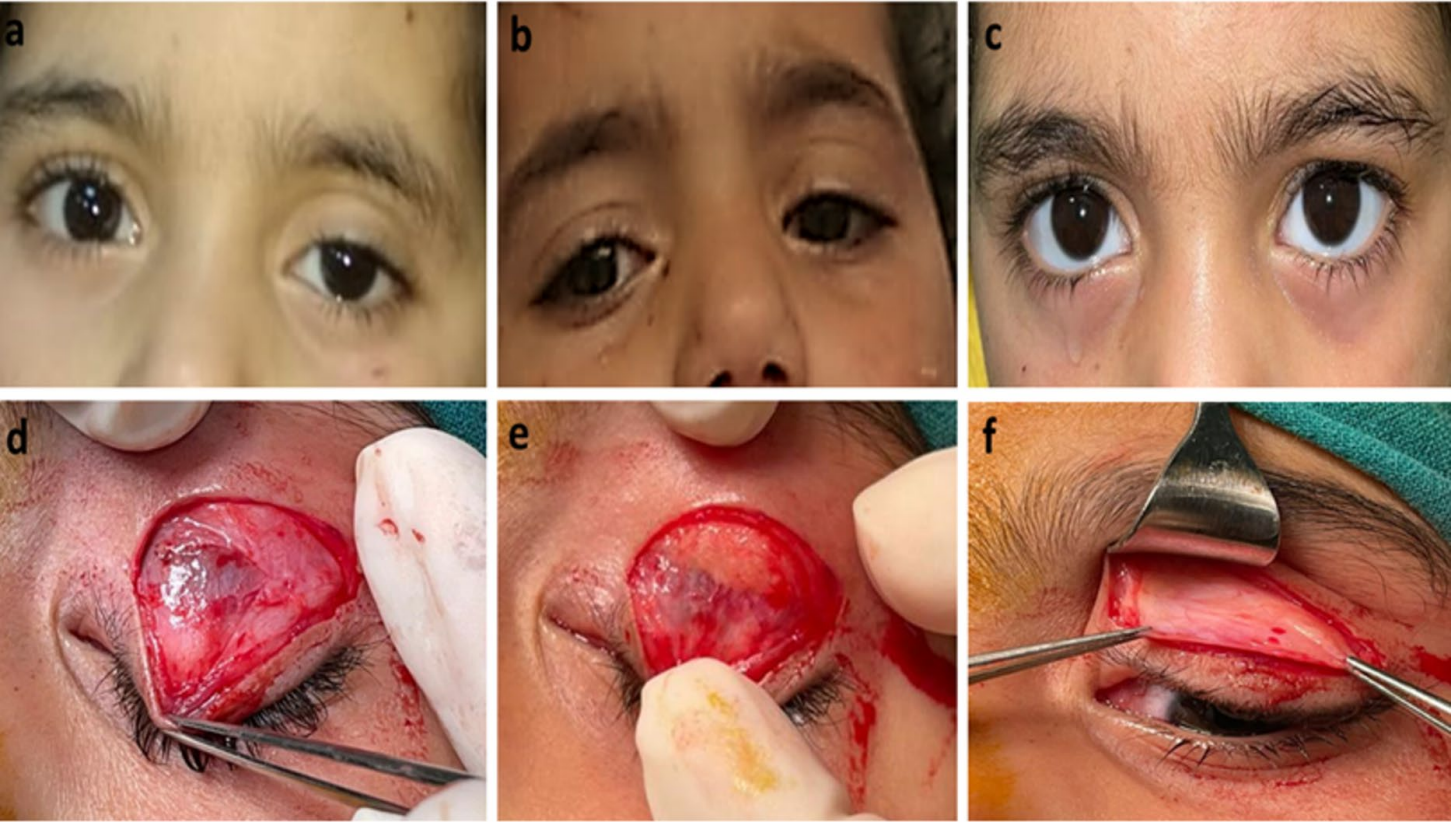
Left congenital ptosis with high crease (**a**), more drooping of the left eye on looking downwards (**b**). A proper postoperative lid position (**c**), Intraoperative dissection shows corneal hue after opening the septum (**d**), bulging of preaponeurotic fat covered by a layer of the septum on pressing on the globe (**e**), then freeing the whole aponeurotic layer (**f**)

**Fig. 2:**
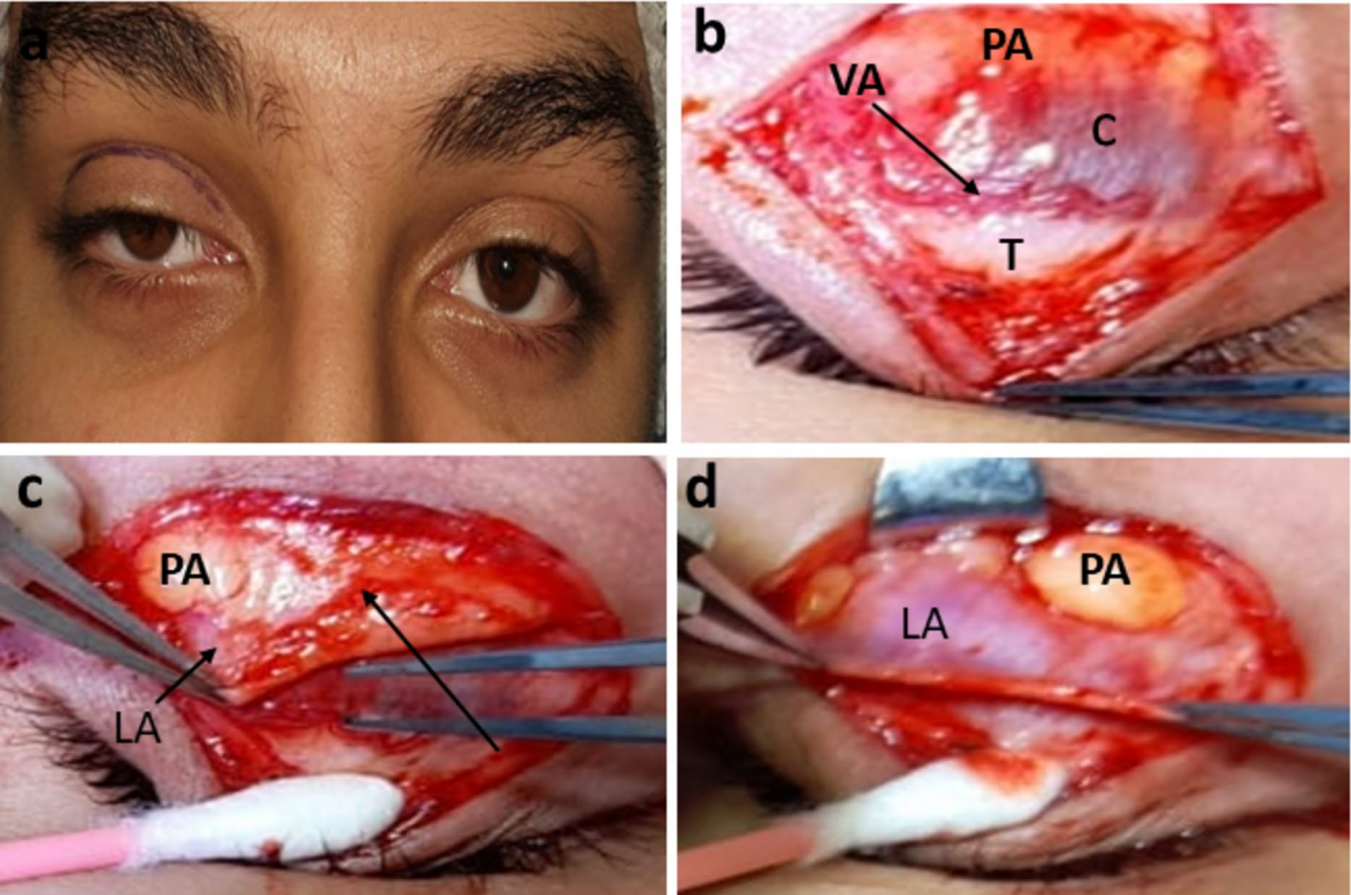
RT congenital ptosis and high crease (**a**). Photo (**b**) shows absent aponeurosis with significant corneal hue (C), prominent vascular arcade (VA) running at the superior border of the tarsus (T). Photo (**c**) shows the edge of the aponeurosis (LA) after cutting through the septal covering of the preaponeurotic fat (PA). Arrow shows point of junction of septum and aponeurosis. Photo (**d**) shows the aponeurosis after complete separation

**Fig. 3:**
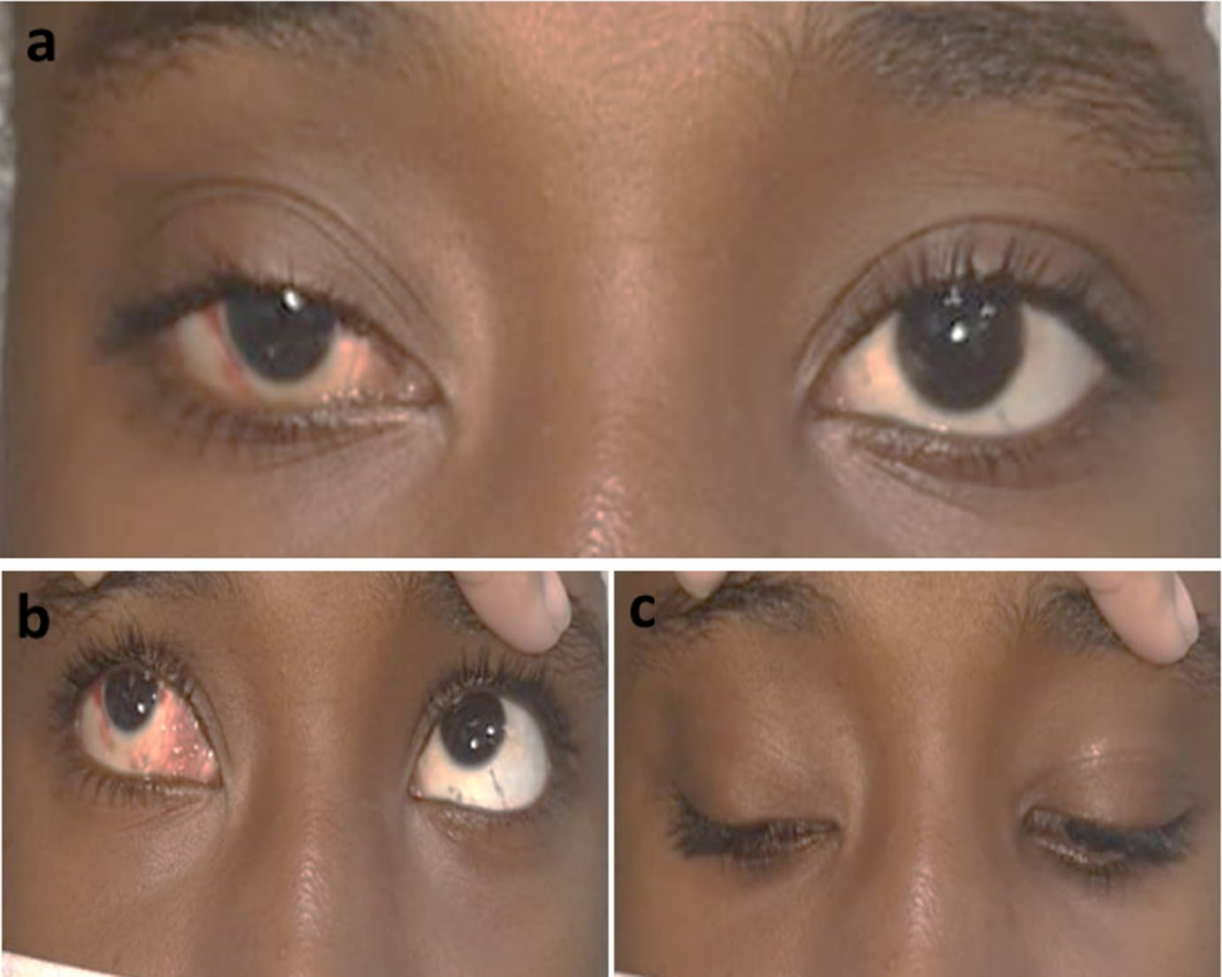
RT congenital ptosis with multiple creases (**a**) good levator function (**b**) with more drooping on looking downwards (**c**)

The preoperative MRD1 of included eyes ranged between −2 to 3 mm (mean 0.44 ± 1.17 mm). The mean preoperative crease height was 10.07 ± 1.62 mm (Figs. 1, 2, and 3), 2 eyes had normal crease height (6 mm) while 25 eyes (92.6%) had a high crease ≥ 8 mm. Multiple creases were recorded in 5 eyes (18.5%) (Fig. 3).

In unilateral cases (70.4%), the lid showed more drooping on the affected side on looking downwards compared to the sound side (Fig. 3). However, Lid lag on down gaze was found in 3 eyes (11.1%), all of them had fair levator function (4 mm).

**Fig. 4:**
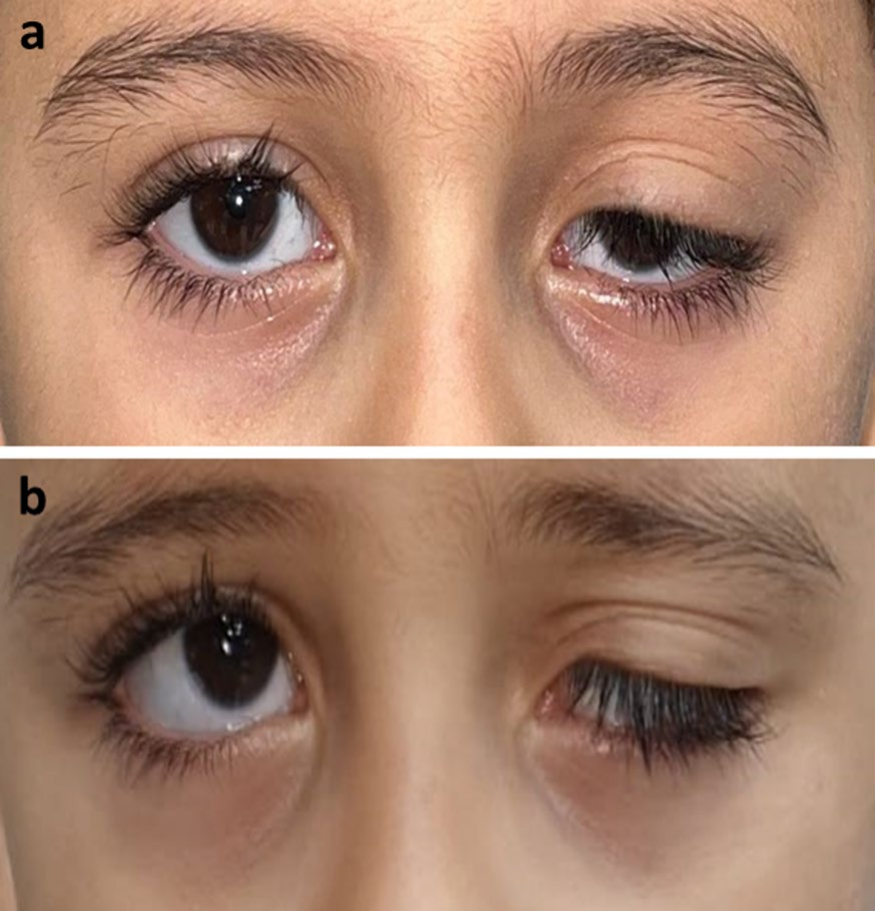
A case with multiple creases poor levator function (**a**), and accentuation of skin fold on attempt of looking upwards (**b**)

The levator function ranged between 4 to 15 mm (mean 8.56 ± 3.89 mm). Three eyes (11.1%) had levator function of 4 mm, and they showed accentuated skin fold above the level of the high crease on attempted up gaze (Fig. 4).

**Fig. 5:**
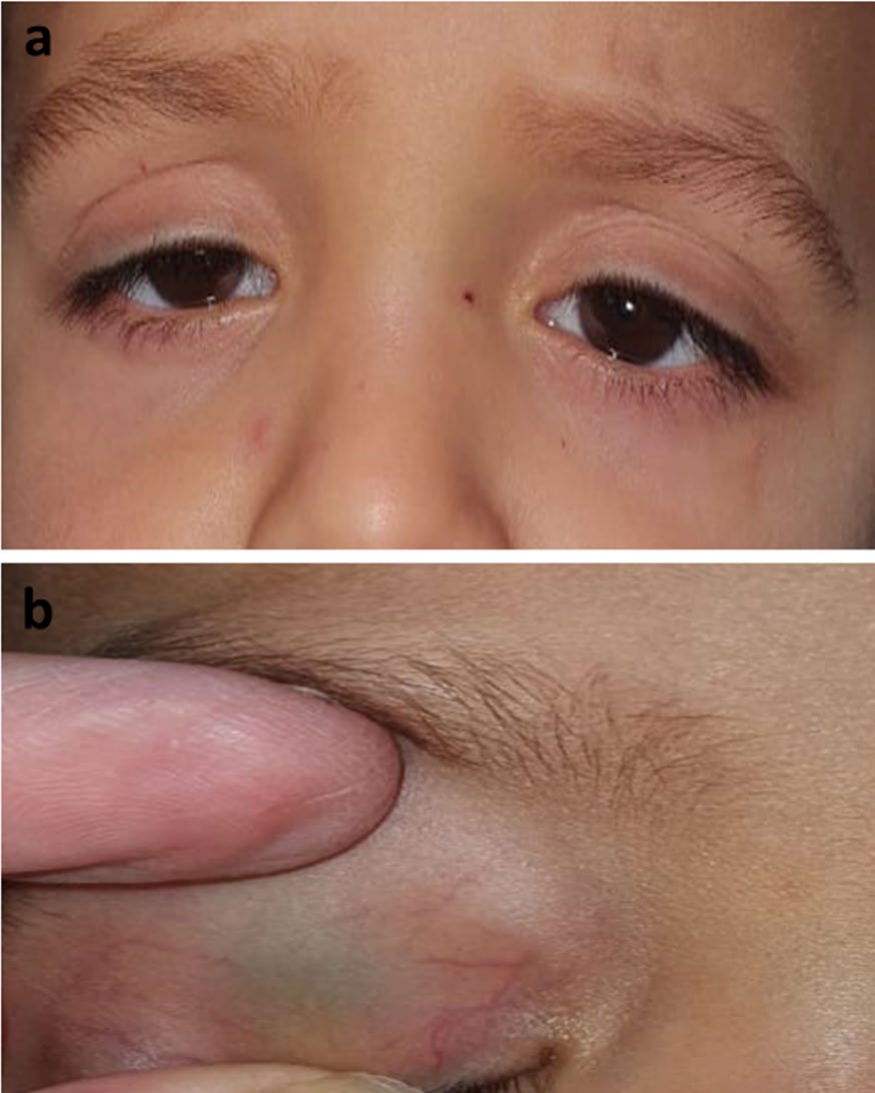
Bilateral congenital ptosis with undefined high creases (**a**). Evident corneal hue through thin lid skin (**b**)

Corneal hue could be seen through the thin lid skin in five eyes of three patients (18.5%) with fair complexion (Fig. 5).

## Intraoperative data

The mean dehiscence distance was 13.44 ± 4.89 mm. Twenty-five eyes out of the twenty-seven met the diagnostic criteria (92.6%) showing total absence of the levator aponeurosis edge at the area above the tarsus. Hence, upon dissecting the preseptal orbicularis muscle and the orbital septum, a bare conjunctival surface was directly found revealing the underlying corneal hue and the blood vessels of the peripheral arcade on the surface of the Muller muscle (confirming the absence of the aponeurotic attachment) (Figs. 1 and 2). Opening of the orbital septum where it covers the distal edge of the preaponeurotic fat resulted in its bulging with the appearance of the “paused edge” of the levator muscle which seemed to fuse with the septum at this point (Fig. 2 and Online Resources 1, 2). However, in two eyes (7.4%), the edge of the aponeurosis was found dehisced just above the superior tarsal border and distal to the fat pad i.e. (achieved septal penetration). Those two eyes had crease height of 6 mm. In the 3 cases with fair Levator function and preoperative lid lag, the retrieved levator muscle was tight and cutting fibrous bands and septal attachments was required to achieve proper mobilization.

The degree of dehiscence was directly correlated to the preoperative crease level (r value 0.25) and inversely related to MRD (r value −0.36). However, this correlation was statistically insignificant (p -value 0.21, 0.066 respectively).

The mean postoperative MRD1 was 4.22 ± 0.75 mm while crease height was 7.07 ± 1.41 mm. Both showed statistically significant change compared to the preoperative values (p value < 0.0001). Two eyes (7.4%) showed under-correction that required surgical exploration revealing suture migration; hence the aponeurosis was reattached.

Based on the reviewed records, 5 patients ( 6 eyes) had been examined after one year of surgical intervention ( for other ophthalmological causes) and were found to have stable surgical outcome.

## Discussion

Aponeurotic defects in senile ptosis are a well-established entity that was first described by Quickert in 1970. It was first described in congenital ptosis by Anderson and Gordy in 1979 who reported criteria like levator disinsertion with good levator function, high lid crease and absence of lid lag. Those criteria are different from those of true congenital ptosis which is mainly due to muscle dysgenesis with a variable degree of levator dysfunction, weak lid crease, downgaze lid lag [5].

They also attributed some of the unsatisfactory results with standard procedures and correct amount of surgery to missing such aponeurotic defects [4]. This entity, however, is less reported in literature [2].

In the current study, criteria of congenital aponeurotic defect were found in 27 eyes out of the reviewed 287 (9.4%). This percentage is higher than that reported primarily by Anderson and Gordy 7% (3 eyes out of 46) [4] and by Martin and Rogers 7.4% (12 out of 162 cases) [2]. The latter authors operated on 14 eyes (12 cases) and the 162 cases were not clearly specified if they were bilateral cases other than the ones they had operated upon in their series. They also included one case of traumatic aponeurotic defect.

Although the amount of ptosis in this study was like both reports (2–5 mm), we believe that the higher percentage could be attributed to larger sample size because of increased awareness of such entity with including wider range of muscle function (4–15 mm). All cases in the above-mentioned reports had good levator function; (8 and 15 mm) in Martin and Rogers’ [2] and (7–10 mm) in Anderson and Gordy’s [4].

The first few cases included in this series were discovered intraoperatively and hence attracted the authors’ attention to such entity. On data reviewing, it was found that there are certain preoperative clues that would be suggestive. These clues included the lid crease position, absence of lid lag on down gaze (except for those found with fair function and intraoperative dense septal adhesions) and the visibility of bluish corneal hue through the thin lid skin.

Absence of lid lag on down gaze was noted in 88.9% of cases with even more drooping compared to the sound side in unilateral cases (14 eyes, 70.4%). Anderson and Gordy described it as a sure sign of congenital aponeurotic defect [4]. We agree on its significance yet, we suggest that its absence does not negate the presence of aponeurotic defect. The lower lid position on the down gaze is suggestive of absent muscle dysgenesis which is present in most congenital myogenic ptosis cases.

The levator function was found to be anywhere from good to fair. This series included three eyes with fair levator function and evident preoperative lid lag, they were scheduled for maximum levator resection as was adopted by the authors. Intraoperatively, all the previously mentioned aponeurotic maldevelopment features were found however, with significant abnormal dense septal fibrous bands especially at the site of the medial and the lateral horns with a totally healthy muscle once freed from the adhesions. These fibrotic changes were found reproducibly in those three cases. All these factors could contribute to the lid lag and probably the cause of the fair function. This hypothesis was raised by some authors in previous reports [6–9]. Some mentioned that the aponeurosis fibrotic changes found in ptotic eyes with poor preoperative levator function could inversely affect its stretching capability and aggravate the limitation of eyelid excursion [10]. Surprisingly simple aponeurotic attachment after complete mobilization of the muscle from its fibrous prison achieved the desired lid position.

The lid skin crease was usually present yet at a higher position compared to the sound side or the expected position in bilateral cases. Twenty-five eyes (92.6%) had a crease at 8 mm or higher compared to 2 eyes (7.3%) with normally positioned crease yet showed an aponeurotic defect intraoperatively aponeurosis was found about 3 mm above the superior tarsal border with a higher septal attachment denoting arrested septal development yet with aponeurosis penetration unlike the rest of cases.

Collins reported that this could happen in the presence of malunion rather than disinsertion [11]. Martin and Rogers also reported that there is always a skin fold/ crease either in its normal position which they attributed to malunion or high which they attributed to disinsertion [2]. In the current study, the three cases with fair levator function had a hardly developed high crease but there was always a high accentuated skin fold on attempted up gaze signifying the upward traction on the paused distal end of the aponeurosis during muscle contraction.

Visibility of the corneal hue through the lid skin is highly suggestive of possible aponeurotic defect and was detected in 18.5% of cases, all had fair complexion which is not common in our community.

Intraoperatively, a sure sign of aponeurotic defect was identifying Muller conjunctival complex with vascular arcade after opening of the orbital septum. As the dissection was carried out meticulously layer by layer [3] and the septum was opened at a higher level than the upper tarsal border, the accidental aponeurosis severance was excluded, and the aponeurosis could only be identified once the preaponeurotic fat was retracted before dissection was carried down to expose the tarsus.

Embryologically, Levator muscle arises from different cell types that migrate to their destination to acquire full maturation [12–14]. Recent literature shows that neural crest cells component of the lid mesenchyme gives rise to levator aponeurosis, orbital septum, and Muller’s muscle while mesodermal component gives rise to striated muscle fibers [15, 16].

The levator muscle starts to arise within the orbit as early as the 6^th^week of embryogenesis. It then acquires its final differentiation at the 10^th^week, and it finally reaches the adult position above the superior rectus at the week thirteen [11].

On the other hand, the aponeurosis starts to appear during the 12^th^week close to the primitive orbital septum, The muscle and the septum come to join below the developing central fat pad around week 20 and reach their final insertion together by week 24 [17].

We presume that any interruption of the downward growth of the septum and aponeurosis between week 12 and week 24 could give rise to this paused development of levator aponeurosis. It also seems that neural crest cell migration defect may have an important role in the pathogenesis of aponeurosis maldevelopment [9].

The dehiscence distance was inversely proportional to MRD 1. This correlation was statistically insignificant (p value: 0.21). This goes with the observation that the degree of ptosis does not necessarily signify the presence of the aponeurotic defect. However, it logically explains the presence of the ptosis due to the inability of the muscle to conduct its action to the tarsus and could barely accentuate the skin fold proximal to the crease on attempted upward gaze due to muscle contraction. This suggests searching for the aponeurosis at a higher level as far as the orbital margin in suspected cases. The amount of dehiscence was also directly related to the crease height yet was statistically insignificant. The absence of properly developed aponeurosis would explain the higher and multiple creases, meanwhile it explains the presence of ptosis; something like aponeurotic ptosis in elderly.

Several techniques and modifications for aponeurosis repair have been reported. Levator muscle advancement and resection are the most popular external techniques used. If total disinsertion is discovered intraoperatively, levator aponeurosis reattachment to the tarsal plate is performed however, if the aponeurosis is stretched and thin but still attached, a resection or advancement may be completed [18, 19].

Internal repair techniques; conjunctival mullerectomy was originally described for patients with mild to moderate ptosis, good levator function, and a positive response to 2.5–5% phenylephrine testing [20].

Simple aponeurosis advancement to the tarsus was done in all cases of our series with no further need for muscle resection, however additional release of the fibrous adhesions was done in the 3 cases with fair levator function for better aponeurosis mobilization.

The desired amount of correction was guided by the gapping method [21, 22]. Even the two re-operated eyes improved by further aponeurotic re-suturing without any muscle shortening.

Simple aponeurosis reinsertion was also done by Martin and Rogers who reported achieving cosmetically good results in (7 out of 12 patients) and reintervention for 5 cases, not emphasizing if these were 7 patients or eyes especially that they had mentioned that they operated on 14 eyes [2].

Despite being an underreported condition, yet a retrospective study with limited number of cases is considered a limitation to truly validate its incidence. A prospective study with a long follow up period is recommended. A future multidisciplinary Collaboration with a geneticist, an embryologist and a developmental biologist is required for a better comprehensive understanding of this developmental anomaly.

Longer scheduled follow-up visits are also recommended in future studies to assess the long-term effect of aponeurotic advancement.

## Conclusion

Congenital aponeurotic maldevelopment is an established yet underreported entity of congenital ptosis. It could be suspected clinically with high crease position, lower lid position on downgaze and presence of corneal hue. This knowledge can improve the surgical procedure, and outcome. Further prospective studies could give an idea about the real incidence of this entity and the range of its clinical presentations.

## Supplementary Information

Below is the link to the electronic supplementary material.

Supplementary file1 (MP4 11338 KB)

Supplementary file2 (MP4 19281 KB)

Supplementary file3 (DOCX 21 KB)

## Data Availability

All data generated or analysed during this study are included in this published article (and its Supplementary Information files).
