# Supplementary material for: Congenital ptosis with aponeurotic maldevelopment: clinical and surgical perspectives: Aponeurotic maldevelopment in congenital ptosis
Source: Int Ophthalmol. 2024 Mar 2;44(1):123. doi: 10.1007/s10792-024-03053-5 (PMC10907453; doi:10.1007/s10792-024-03053-5)
Supplement: Supplementary file 3 — Supplementary file3 (DOCX 21 KB) [file 10792_2024_3053_MOESM3_ESM.docx]

| Patient no | Age  (y) | sex | side | Preop.  MRD1  (mm) | Preop.  Crease  (mm) | Levator function  (mm) | Degree of dehiscence  (mm) | Preop.  Lid lag | Postop.  MRD1  (mm) | Postop.  Crease  (mm) |
| --- | --- | --- | --- | --- | --- | --- | --- | --- | --- | --- |
| 1 | 9 | m | LT | 0.5 | 12 | 12 | 13 | no | 3 | 8 |
| 2 | 17 | f | LT | 0 | 10 | 10 | 15 | no | 4 | 8 |
| 3 | 6 | m | RT | 2 | 10 | 14 | 10 | no | 4 | 6 |
| 4 | 3 | f | LT | 2 | 9 | 15 | 9 | no | 5 | 5 |
| 5 | 5 | f | LT | 1 | 10 | 12 | 11 | no | 4 | 6 |
| 6 | 18 | m | RT | -1 | 12 | 13 | 15 | no | 4 | 8 |
| 7 | 8 | m | LT | 0 | 6 | 10 | 6 | no | 3 | 6 |
| 8 | 25 | f | RT | -1 | 10 | 7 | 18 | no | 5 | 8 |
| 9 | 8 | f | LT | 0 | 12 | 5 | 12 | no | 4 | 7 |
| 10 | 9 | f | LT | 3 | 6 | 12 | 6 | no | 5 | 5 |
| 11 | 3 | f | LT | 0.5 | 10 | 4 | 10 | yes | 5 | 6 |
| 12 | 5 | m | RT | -2 | 8 | 5 | 20 | no | 4 | 6 |
| 13 | 4 | f | LT | -2 | 10 | 4 | 18 | yes | 4 | 6 |
| 14 | 12 | f | RT | 1 | 12 | 15 | 10 | no | 5 | 5 |
| 15 | 1 | f | RT | 1 | 10 | 5 | 25 | no | 3 | 8 |
|  | 1 | f | LT | 1 | 10 | 5 | 25 | no | 5 | 6 |
| 16 | 3 | f | LT | 1 | 9 | 7 | 12 | no | 4 | 9 |
| 17 | 5 | m | RT | 0 | 9 | 5 | 8 | no | 5 | 8 |
| 18 | 17 | f | RT | 1 | 12 | 8 | 15 | no | 4 | 10 |
|  | 17 | f | LT | 0 | 11 | 8 | 15 | no | 4 | 10 |
| 19 | 14 | m | RT | 1 | 10 | 8 | 8 | no | 4 | 8 |
|  | 14 | m | LT | -1 | 10 | 5 | 12 | no | 3 | 8 |
| 20 | 4 | m | RT | 0 | 10 | 4 | 15 | yes | 5 | 7 |
|  | 4 | m | LT | 1 | 10 | 5 | 15 | no | 5 | 6 |
| 21 | 16 | m | RT | 1 | 12 | 15 | 10 | no | 5 | 7 |
| 22 | 14 | f | RT | 0 | 12 | 7 | 15 | no | 3 | 8 |
|  | 14 | f | LT | 2 | 10 | 12 | 15 | no | 5 | 6 |
| Mean value | 9.48 ± 6.35 |  |  | 0.44 ± 1.17 | 10.07 ± 1.62 | 8.56 ± 3.89 | 13.44 ±  4.89 |  | 4.22 ± 0.75 | 7.07 ±  1.41 |

Table (1) Demographic, preoperative, intraoperative, and postoperative data of the 22 patients
